# Use of dietary supplements by cardiologists, dermatologists and orthopedists: report of a survey

**DOI:** 10.1186/1475-2891-10-20

**Published:** 2011-03-03

**Authors:** Annette Dickinson, Andrew Shao, Nicolas Boyon, Julio C Franco

**Affiliations:** 1 Dickinson Consulting, LLC, 3432 Denmark Avenue, #350, St. Paul, MN 55123, USA; 2 Scientific and Regulatory Affairs, Council for Responsible Nutrition, 1828 L Street, N.W., Suite 510, Washington, D.C. 20036, USA; 3 Ipsos Public Affairs, 1700 Broadway, New York, NY 10019, USA

## Abstract

**Background:**

Dietary supplements are regularly used by a majority of the American population, and usage by health professionals is also common. There is considerable interest in usage patterns within the population and in the reasons for using dietary supplements. The "Life...supplemented" Healthcare Professionals 2008 Impact Study (HCP Impact Study) surveyed usage of dietary supplements by physicians in three specialties: cardiology, dermatology, and orthopedics.

**Methods:**

The HCP Impact Study was conducted online by Ipsos Public Affairs for the Council for Responsible Nutrition (CRN), a trade association of the dietary supplement industry. Respondents were 900 physicians, including 300 each from three specialties - cardiology, dermatology, and orthopedics.

**Results:**

Fifty-seven percent of cardiologists said they use dietary supplements at least occasionally, as did 75% of dermatologists and 73% of orthopedists. The product most commonly reported to be used was a multivitamin, but over 25% in each specialty said they used omega-3 fatty acids and over 20% said they used some botanical supplements. Regular dietary supplement use was reported by 37% of cardiologists, 59% of dermatologists, and 50% of orthopedists. Seventy-two percent of cardiologists, 66% of dermatologists, and 91% of orthopedists reported recommending dietary supplements to their patients. The primary reason given for recommending dietary supplements to patients was for heart health or lowering cholesterol for the cardiologists; benefits for skin, hair and nails for the dermatologists; and bone and joint health for the orthopedists.

**Conclusions:**

Reported dietary supplement use was relatively common in this sample of physicians, and when they recommended dietary supplements to patients, they tended to do so for reasons related to their specialty.

## Background

Most American adults use dietary supplements, as documented by numerous surveys, including the National Health and Nutrition Examination Survey (NHANES) and the National Health Interview Survey (NHIS) [1-4]. Recognizing that nutrient shortfalls are common in U.S. diets, the 2005 *Dietary Guidelines for Americans *and the recommendations of groups such as the American Dietetic Association and the National Osteoporosis Foundation emphasize improving food habits but also recognize that dietary supplements can play an important role [5-7]. The 2010 *Dietary Guidelines for Americans *identify seven "nutrients of concern" because of low intakes in the general population or in some population groups and recognize that "in certain cases, fortified foods and dietary supplements may be useful in providing one or more nutrients that otherwise might be consumed in less than recommended amounts." [8] Some experts have recommended the routine use of a multivitamin for many adults, in order to ensure adequate nutrient intakes of a variety of vitamins and minerals [9,10]. Calcium and vitamin D are critical to ensuring bone health and may also impact other health conditions, and shortfalls in intake are widespread [11,12]. Supplemental intakes of the omega-3 fatty acids EPA and DHA have been shown to improve cardiovascular health and may also confer other health benefits [13]. Some but not all studies indicate that specialty products such as glucosamine and chondroitin sulfate may have a favorable impact on the health of the joints [14,15]. Botanical products may have a variety of health benefits, and the Office of Dietary Supplements at NIH, in cooperation with the National Center for Complementary and Alternative Medicine (NCCAM), has funded numerous academic research centers to undertake further research in this area [16].

Health professionals including physicians have an interest in healthy lifestyles and in habits that may contribute to wellness, potentially including the use of dietary supplements. Some surveys of physicians suggest that they are as likely as members of the general public to use dietary supplements [17-20]. We have previously reported on a 2007 survey of dietary supplement use in physicians and nurses [21]. We now report the findings of a 2008 survey of the use of dietary supplements by cardiologists, orthopedists, and dermatologists, and the extent to which they recommend dietary supplements to their patients.

## Methods

The Council for Responsible Nutrition (CRN, Washington, DC), a trade association representing the dietary supplement industry, contracted with Ipsos Public Affairs (New York, NY) in 2008 to conduct a "Life...supplemented" Healthcare Professionals Impact Study (HCP Impact Study) -- the second of a series of surveys of health professionals regarding their dietary supplement use and whether they recommend dietary supplements to patients. "Life...supplemented" is a consumer wellness initiative funded by a number of CRN member companies [22].

The survey questionnaire was administered online between August 28 and September 12, 2008, to 900 physicians, including 300 cardiologists, 300 dermatologists, and 300 orthopedists. The sample size for each specialty was selected to provide a 95% confidence level that the margin of sampling error for the estimates is no greater than plus-or-minus 5.7%.

To protect against conflicts of interest, all physicians surveyed were screened to ensure that none of them were affiliated with a market research company or advertising agency, a medical education company, or a pharmaceutical or dietary supplement company. Additional qualifications for participation in the current survey included being a board-certified or board-eligible cardiologist, dermatologist, or orthopedist working primarily in an outpatient practice and currently seeing patients in their office each week. Recruitment was halted when 300 subjects in each specialty had completed the survey.

The 900 physicians surveyed consisted of 198 members of the eRewards U.S. online physician panel (53 cardiologists, 89 dermatologists and 56 orthopedists) and another 702 physicians (247 cardiologists, 211 dermatologists and 244 orthopedists) from a database provided by the American Medical Association (AMA, Chicago, IL).

The eRewards U.S. online physician panel includes over 36,000 physicians who were initially recruited by telephone, mail, fax or email, and who "opted into" the panel in order to take part in market research surveys. For a fee, market research firms such as Ipsos and other organizations such as pharmaceutical companies can obtain access to eRewards' physician panel and invite them via email to take part in online surveys without violating anti-spamming laws. A total of 1,376 cardiologists, dermatologists, and orthopedists in the eRewards panel were sent an email invitation to take part in the survey. As compensation for their participation, they were offered an honorarium ranging between $27 and $31. A total of 412 physicians responded. The response rate of 30% was well within the norms for online surveys conducted among eRewards physician panel members from these specialties. Among those who responded, 48% met all qualifications and completed the entire survey. The remainder consisted of those who did not meet all qualifications necessary to take part in the survey (46%); those who met the desired qualifications, but failed to complete the entire questionnaire (5%); and those who did not take part in the survey because the target number of completed interviews had been achieved (1%).

To complement the sample, a comprehensive database from the AMA listing 800,000 physicians was used. A total of 35,095 randomly selected, pre-qualified cardiologists, dermatologists, and orthopedists were sent an invitation to take part in the survey--either by email if they had expressly authorized being contacted this way, by direct mail, or by fax. As compensation for their participation, they were offered an honorarium ranging between $33 and $57. A total of 1,187 physicians responded. The response rate of 3% was typical of online surveys among specialists sourced from the AMA-supplied database and contacted by email, direct mail or fax. Among those who responded, 59% met all qualifications and completed the entire survey, while 24% were excluded because the target number of completed interviews had been achieved, 14% did not meet all the qualifications needed to take part in the survey, and 3% met the desired qualifications, but failed to complete the entire questionnaire.

Respondents in this survey were physicians who volunteered to participate in this particular survey relating to dietary supplements. While some of these respondents may be self-selected healthcare professionals with a given interest in dietary supplements, that interest could be positive or negative. Also, respondents were informed of its topic only once they accessed the survey, as there was no reference to dietary supplements in the invitation letter.

In the survey instrument, dietary supplements were defined to include a broad spectrum of products: vitamins, minerals, herbals, botanicals, sports nutrition, or specialty supplements. Respondents were asked to select one term that best described their use of dietary supplements: regular use of a variety of dietary supplements, regular use typically including only a multivitamin or multivitamin-mineral product, occasional use, seasonal use, used in the past but no longer, or never used dietary supplements. Regular use was not specifically defined in the survey instrument, except indirectly in contrast to the other terms. Occasional use was defined as taking supplements "throughout the year when I think of it or when the need arises." Seasonal use was defined as "taking them only during part of the year such as during the cold/flu season or allergy season."

Respondents who said they used dietary supplements and respondents who said they recommended dietary supplements to patients were asked to indicate their reasons for doing so. They were asked to choose all relevant responses from a list of 29 potential reasons, including: allergies; anti-aging; anxiety; bone health; cognitive function/memory; depression; dietary pattern (vegetarian); digestive/gastrointestinal health; energy; eye health; flu/colds; headaches; heart health; immune health; insomnia; joint health; lower cholesterol; maintain healthy cholesterol; men's health (prostate health or BPH); men's health (sexual function); musculoskeletal pain; overall health/wellness benefits; skin, hair and nails; sports performance; weight management; women's health: menopause; women's health: painful periods or premenstrual syndrome; women's health: prenatal/pregnancy; or women's health: other. They were also given the option of specifying a reason other than those included in the list.

## Results

Over 90% of the cardiologists and orthopedists and almost 70% of the dermatologists who participated in this survey were male. Most were in the 40-to-59 age range, including 71% of the cardiologists, 55% of the dermatologists, and 61% of the orthopedists. Most had been qualified in their specialty for more than 10 years -- 67% of the cardiologists, 65% of the dermatologists, and 70% of the orthopedists. Geographically, the samples were well dispersed. (Data not shown.)

Among the physicians who took part in the survey, 57% of the cardiologists, 75% of the dermatologists, and 73% of the orthopedists reported using dietary supplements regularly, occasionally, or seasonally. More precisely, regular use of dietary supplements was reported by 37% of cardiologists, 59% of dermatologists, and 50% of orthopedists, and occasional or seasonal usage was reported by 21% of cardiologists, 16% of dermatologists and 22% of orthopedists. In addition, 18% of cardiologists, 8% of dermatologists, and 11% of orthopedists said they had taken supplements in the past but no longer considered themselves supplement users. Twenty-five percent of cardiologists, 17% of dermatologists, and 16% of orthopedists said they had never taken dietary supplements. For a summary of the main results, see Table 1.

**Table 1:** Use and recommendation of dietary supplements by cardiologists, dermatologists, and orthopedists in the HCP Impact Study

	Cardiologists (n = 300)	Dermatologists (n = 300)	Orthopedists (n = 300)
Percentage using dietary supplements (regularly, occasionally, seasonally)	57%	75%	73%

Percentage of *regular *users	37%	59%	50%

Percentage recommending dietary supplements to patients	72%	66%	91%

Regular supplement users were asked whether they typically take *only *a multivitamin or whether they take a variety of products. Multivitamins may also be included within the "variety of products" category. More cardiologists and dermatologists said they regularly use only a multivitamin than said they regularly use a variety of supplements (22% vs. 15%, respectively, among cardiologists, and 34% vs. 25% among dermatologists). However, proportions of regular multivitamin-only users and regular users of a variety of supplements were not significantly different among orthopedists (24% vs. 27%).

Most of the physicians who reported supplement use in this survey were long-term users. About half of the supplement users in each specialty said they had taken supplements for 4 to 10 years, and about a third said they had taken supplements for more than 10 years. Physicians who said they used dietary supplements were asked to select among 29 possible reasons those for which they specifically do so. The reason most commonly given for taking dietary supplements was overall health and wellness, selected by 32% of cardiologists, 42% of dermatologists and 43% of orthopedists. Heart health was mentioned by more than a quarter of cardiologists and orthopedists, while bone health was mentioned by about a quarter of orthopedists and dermatologists. Lowering cholesterol was cited by 20% of cardiologists, joint health by 29% of orthopedists, and skin, hair and nails by 16% of dermatologists.

Among 26 listed types of dietary supplement products, the one most commonly reported to be used was the multivitamin. In our survey, 44% of all cardiologists, 61% of all dermatologists, and 57% of all orthopedists said they had used a multivitamin within the past year, and many also used additional products. Over 25% of physicians in each specialty said they had used omega-3/fish oil supplements, and over 20% of each of the three specialty groups said they had taken a botanical supplement in the past year, with green tea being the botanical most often mentioned. Vitamin C, calcium, omega-3 or fish oil, and glucosamine/chondroitin sulfate were mentioned by at least 20% of at least one physician specialty.

In this survey, the physicians were asked, "Do you ever recommend dietary supplements to patients?" A large proportion of the physicians surveyed said they recommended supplements to their patients -- 72% of cardiologists, 66% of dermatologists, and 91% of orthopedists. Among those who said they use supplements themselves, the proportion who also said they recommended supplements to their patients was slightly higher (86% of cardiologists, 79% of dermatologists, and 94% of orthopedists).

Physicians who ever recommend dietary supplements to patients were asked to select all the reasons why they do so. The list was the same one that had been used to ask physicians who personally take supplements why they do so. The reasons given by physicians for recommending dietary supplements to patients were somewhat different from those given for personally using such products. While overall health and wellness was the primary reason given for personal use, the reasons given for recommending dietary supplements to patients were clearly related to the physicians' specialty. Among cardiologists, lowering cholesterol, maintaining healthy cholesterol, and general heart health were cited more often than overall wellness as reasons for recommending dietary supplements. Among dermatologists, 81% of those who said they recommended dietary supplements named reasons related to skin, hair, and nails, compared to only 30% citing overall wellness. Among orthopedists, 75% of those who said they recommended dietary supplements mentioned bone health, 73% mentioned joint health, and 53% mentioned musculoskeletal pain as the reason for their recommendation, while only 25% mentioned overall wellness. **See **Table 2 for a comparison of the top reasons for using *vs*. the top reasons for recommending dietary supplements, for each specialty.

**Table 2:** Primary reasons for taking and recommending dietary supplements by cardiologists, dermatologists, and orthopedists in the HCP Impact Study*

SPECIALTY	REASONS FOR TAKING	REASONS FOR RECOMMENDING
CARDIOLOGISTS:	(n = 172 who take supplements)	(n = 217 who recommend supplements)
Overall health/wellness	**32%**	**30%**
Heart health	**29%**	**55%**
Lower cholesterol	**20%**	**58%**
Bone health	**16%**	**22%**
Joint health	**16%**	19%
Maintain healthy cholesterol	15%	**36%**

DERMATOLOGISTS:	(n = 225 who take supplements)	(n = 198 who recommend supplements)
Overall health/wellness	**42%**	**30%**
Bone health	**24%**	**25%**
Skin, hair, nails	**16%**	**81%**
Heart health	**15%**	**12%**
Anti-aging	**14%**	**20%**
Immune health	13%	**12%**

ORTHOPEDISTS:	(n = 218 who take supplements)	(n = 272 who recommend supplements)
Overall health/wellness	**43%**	**25%**
Joint health	**29%**	**73%**
Heart health	**26%**	10%
Bone health	**25%**	**75%**
Flu/colds	**22%**	7%
Musculoskeletal pain	17%	**53%**
Lower cholesterol	16%	**13%**

* Figures in bold show each specialty's top 5 reasons for taking dietary supplements and top 5 reasons for recommending dietary supplements to patients.

Most physicians surveyed (96% of cardiologists, 89% of dermatologists and 97% of orthopedists) said they discussed dietary supplements with their patients. Among them, nearly all (97% of the cardiologists, 94% of the dermatologists and nearly 100% of the orthopedists who reported having such discussions) said that their patients ask them about specific dietary supplements. The particular products the physicians mentioned being asked about by their patients tend to be related to their own specialty. Cardiologists said their patients most often ask about omega-3/fish oil, coenzyme Q10, or vitamin E. Dermatologists say their patients most often ask about vitamin E, vitamin D, and omega-3/fish oil. And orthopedists cite glucosamine/chondroitin, calcium, and vitamin D as those their patients ask about most. **See **Table 3 for more information on the products specialists are most asked about by patients who visit them.

**Table 3:** Top three dietary supplements cardiologists, dermatologists or orthopedists in the HCP Impact Study say their patients ask them about

SPECIALTY AND PRODUCTS	PERCENT WHO SAY PATIENTS ASK ABOUT SPECIFIC PRODUCT
CARDIOLOGISTS	(n = 288 who discuss supplements with patients)
Omega-3/fish oil	80%
Coenzyme Q10	58%
Vitamin E	39%

DERMATOLOGISTS	(n = 266 who discuss supplements with patients)
Vitamin E	45%
Vitamin D	42%
Omega-3/fish oil	41%

ORTHOPEDISTS	(n = 290 who discuss supplements with patients)
Glucosamine/chondroitin	86%
Calcium	63%
Vitamin D	43%

When probed about their attitudes toward dietary supplement use, 55% of cardiologists, 69% of dermatologists, and 75% of orthopedists agreed with the statement, "It is a good idea for patients to take multivitamins." Also, 69% of cardiologists agreed with the statement, "Adults with a family history of heart disease should consider taking dietary supplements containing omega-3 fatty acids/fish oil." And 93% of orthopedists agreed with the statement, "Adults with a family history of osteoporosis or poor bone health should consider taking a calcium dietary supplement."

Most physicians said professional journals and clinical studies were their most trusted source for reliable information about dietary supplements. Most indicated they had not received any formal education or training on the subject of dietary supplements, but two in three physicians across all three specialties said they would be interested in Continuing Medical Education regarding these products. Those physicians expressed an interest in information about products related to their specialty; basic facts about dietary supplements; potential interactions with pharmaceuticals; how to counsel patients about dietary supplements; and medical, legal and ethical issues relating to dietary supplements.

## Discussion

The level of usage of dietary supplements reported by physicians in this survey is similar to the level of usage reported in some surveys of the general population. However, it should be noted that the reported level of usage in the general population is not consistent across surveys, in part because the exact nature of the question asked varies among surveys. Some surveys inquire about use within a short period of time such as the past two weeks or month, some ask about use within the past year, and some pose a general question about supplement use without specifying a time period. The first of these approaches will capture primarily regular users as well as some occasional users, while the last approach will capture virtually all supplement users. Some surveys inquire only about vitamin/mineral supplement use and some cover a broader range of dietary supplements.

The HCP Impact Study asked whether respondents took dietary supplements regularly, occasionally, seasonally, in the past, or never. The question was not limited to vitamin/mineral supplements, but included all types of dietary supplements. The prevalence of *regular *dietary supplement use reported by dermatologists and orthopedists in this survey (59% and 50%, respectively) was similar to the prevalence of use reported among adults in NHANES 1999-2000 and NHANES 2003-2006, where 52% and 54% of adults surveyed said they had taken supplements in the past month [1,2]. In the NHANES 1999-2000 study, the prevalence of supplement usage was higher in subgroups of adults more nearly comparable to the physicians in the HCP Impact Study, in terms of age and education: usage was 56% among adults in the 40-to-60 age range and 62% among adults with more than a high school education. NHANES 2003-2006 and NHIS 2000 also reported increased levels of supplement use in adults with more education and in older adults compared to young adults, although different age groupings were used [2,3]. In the HCP Impact Study, the prevalence of regular dietary supplement use by cardiologists was only 37% -- lower than for the other physician specialists and lower than for the general population in recent NHANES reports.

The proportion of physicians in the HCP Impact Study who reported any supplement use is relatively high, since this figure includes seasonal or occasional use, as well as regular use. Our total usage figure was 75% for dermatologists and 73% for orthopedists, while total usage for cardiologists was lower at 57%. For comparison, a survey of a large multiethnic cohort reported that 58% of men and 72% of women used any of eight dietary supplements regularly (at least once a week) [23]. It should be noted, however, that the multiethnic survey included as supplement users only those who used one of the defined eight product types at least once a week and therefore did not include seasonal or occasional users and did not include users of products other than the eight specified.

A series of consumer surveys conducted for the Council for Responsible Nutrition also provide information about dietary supplement use in the general population. In a survey of about 2000 individuals in 2009, 65% of respondents identified themselves as dietary supplement users (regular, occasional, or seasonal). This figure has been very consistent in surveys conducted by CRN over the past several years: 64% in 2008, 68% in 2007, and 66% in 2006 [24].

Other surveys of dietary supplement use among health professionals have reported substantial levels of usage, from 64% to over 80%. In a survey of women physicians, it was reported that 64% used vitamin or mineral supplements at least occasionally, and 47% of the women used a vitamin or mineral supplement at least 5 days a week [18]. Two surveys of health professionals enrolled in an online course on dietary supplements reported high levels of supplement use (over 80%), perhaps reflecting the interest that led them to enroll in the course [19,20].

We have previously reported the results of an earlier survey of dietary supplement use by physicians and nurses [21]. In that survey, 72% of the physicians and 89% of the nurses said they used dietary supplements at least occasionally, while 51% of the physicians and 59% of the nurses said they were regular supplement users. The current results for dermatologists and orthopedists are similar to our earlier findings for other physicians, but reported usage by cardiologists in the current survey is lower.

The use of dietary supplements can be viewed as one of several elements of a healthy lifestyle, since the use of dietary supplements is associated with the adoption of other healthy habits, including control of body weight, engaging in moderate or vigorous physical activity, and not smoking [1]. The vast majority of the physicians included in this survey said they tried to eat a balanced diet (80% of cardiologists, 81% of dermatologists, and 77% of orthopedists), maintain a healthy weight (69%, 79%, and 69%, respectively), and exercise regularly (67%, 70%, and 69%, respectively).

It is interesting that the reasons given for using dietary supplements and the reasons given for recommending dietary supplements to patients were not the same. Maintaining wellness was the top reason for personal use, but reasons relating to specific health issues were the top reasons for patient recommendations, and the health issues were related to the specialty of the physicians. This is consistent with the fact that the type of supplement most commonly used by physicians is a multivitamin, while the supplements they discuss most with their patients reflect their specialty. This is not surprising, since their patients are people who have come to a medical specialist, presumably with a specific problem, and the physicians make recommendations for medications, interventions, or possibly dietary supplements within their field of expertise. Cardiologists were more likely to say they made recommendations related to heart health and cholesterol; dermatologists, to the condition of the skin, hair and nails; and orthopedists, to bone and joint health or musculoskeletal pain.

Although majorities of physicians included in our survey reported using and recommending supplements, they clearly were also aware of potential concerns, as indicated by their interest in continuing education regarding issues such as interactions with pharmaceuticals. There has been a longstanding concern that medical education fails to provide practitioners with a sound basis for evaluating the role of nutrition in health and disease [25]. Providing more nutrition education in medical schools and increasing the availability of Continuing Medical Education relating to nutrition, including discussion of the role of dietary supplements, would be beneficial for physicians as well as for their patients.

## Conclusions

The HCP Impact Study shows that physician specialists are very likely to use dietary supplements (57 to 75%) and also shows that most of them may recommend dietary supplements to their patients (66 to 91%). Their reasons for recommending dietary supplements are related to their specialty and differ from their reasons for using dietary supplements themselves. Most physicians in this survey indicated that they had not received any formal education or training on the subject of dietary supplements and expressed an interest in Continuing Education regarding these products. There is a need for expanded medical education regarding the general topic of nutrition as well as the more specific topic of dietary supplements.

## Abbreviations

CRN: Council for Responsible Nutrition; HCP Impact Study: "Life...supplemented" Healthcare Professionals Impact Study; NHANES: National Health and Nutrition Examination Survey

## Competing interests

AD is a consultant to the Council for Responsible Nutrition (CRN), and was formerly a VP and President of the association. AS is SVP of CRN. NB is SVP and JCF is Senior Research Manager for Ipsos Public Affairs, which conducted the survey for CRN.

## Authors' contributions

AD prepared the original draft of the article, for subsequent evaluation and elaboration by all of the authors working collaboratively. NB and JF participated in the design and administration of the survey, including the data analysis. All of the authors provided meaningful insight regarding the results and implications of the survey findings, in the context of previously reported research, and all approved the final version of the article.

## References

[B1] Radimer K Bindewald B Hughes J Ervin B Swanson C Picciano MF Dietary supplement use by US adults: data from the National Health and Nutrition Examination Survey, 1999-2000 Am J Epidemiol 2004 160 4 339 349 10.1093/aje/kwh207 15286019

[B2] Bailey RL Gahche JJ Lentino CV Dwyer JT Engel JS Thomas PR Betz JM Sempos CT Picciano MF Dietary Supplement Use in the United States, 2003-2006 J Nutr 2011 141 2 261 6 Epub 2010 Dec 22 10.3945/jn.110.133025 21178089 PMC3021445

[B3] Fennell D Determinants of supplement usage Prev Med 2004 39 5 932 939 10.1016/j.ypmed.2004.03.031 15475026

[B4] Millen AE Dodd KW Subar AF Use of vitamin, mineral, nonvitamin, and nonmineral supplements in the United States: The 1987, 1992, and 2000 National Health Interview Survey results J Am Diet Assoc 2004 104 6 942 950 10.1016/j.jada.2004.03.022 15175592

[B5] Department of Agriculture and Department of Health and Human Services Dietary Guidelines for Americans 2005 Sixth Washington, D.C.: Government Printing Office

[B6] Marra MV Boyar AP Position of the American Dietetic Association: nutrient supplementation J Am Diet Assoc 2009 109 12 2073 2085 10.1016/j.jada.2009.10.020 19957415

[B7] Osteoporosis: fast facts. Prevention: calcium. Prevention: vitamin D http://www.nof.org

[B8] Department of Agriculture and Department of Health and Human Services Dietary Guidelines for Americans 2010 Seventh Washington, D.C.: Government Printing Office 10.3945/an.111.000430 PMC3090168 22332062

[B9] Willett WC Bendich A, Deckelbaum RJ Potential benefits of preventive nutrition strategies Preventive Nutrition: The Comprehensive Guide for Health Professionals 2005 Third Totowa, NJ: Humana Press

[B10] Fletcher RH Fairfield KM Vitamins for chronic disease prevention in adults: clinical applications J Am Med Assn 2002 287 23 3127 3129 10.1001/jama.287.23.3127 12069676

[B11] Department of Health and Human Services Bone health and osteoporosis: A report of the Surgeon General 2004 Washington, D.C.: U.S. Government Printing Office

[B12] Holick MF High prevalence of vitamin D inadequacy and implications for health Mayo Clin Proc 2006 81 3 353 373 10.4065/81.3.353 16529140

[B13] Harris WS Mozaffarian D Lefevre M Toner CD Colombo J Cunnane SC Holden JM Klurfeld DM Morris MC Whelan J Towards establishing dietary reference intakes for eicosapentaenoic and docosahexaenoic acids J Nutr 2009 139 4 804S 819S 10.3945/jn.108.101329 19244379 PMC6459058

[B14] Clegg DO Reda DJ Harris CL Klein MA O'Dell JR Hooper MM Bradley JD Bingham CO Weisman MH Jackson CG Glucosamine, chondroitin sulfate, and the two in combination for painful knee osteoarthritis N Engl J Med 2006 354 8 795 808 10.1056/NEJMoa052771 16495392

[B15] GAIT Trial The NIH Glucosamine/Chondroitin Arthritis Intervention Trial (GAIT) J Pain Palliat Care Pharmacother 2008 22 1 39 43 10.1080/15360280801989351 28792807

[B16] Swanson CA Liu QY Introduction to the National Institutes of Health Botanical Research Centers program Am J Clin Nutr 2008 87 2 471S 18258640 10.1093/ajcn/87.2.471S

[B17] Spencer EH Bendich A Frank E Vitamin and mineral supplement use among US medical students: A longitudinal study J Am Diet Assoc 2006 106 12 1975 1983 10.1016/j.jada.2006.09.003 17126627

[B18] Frank E Bendich A Denniston M Use of vitamin-mineral supplements by female physicians in the United States Am J Clin Nutr 2000 72 4 969 975 11010939 10.1093/ajcn/72.4.969

[B19] Gardiner P Woods C Kemper KJ Dietary supplement use among health care professionals enrolled in an online curriculum on herbs and dietary supplements BMC Complement Altern Med 2006 6 21 10.1186/1472-6882-6-21 16768802 PMC1526756

[B20] Kemper KJ Gardiner P Woods C Changes in use of herbs and dietary supplements (HDS) among clinicians enrolled in an online curriculum BMC Complement Altern Med 2007 7 21 10.1186/1472-6882-7-21 17565695 PMC1904461

[B21] Dickinson A Boyon N Shao A Physicians and nurses use and recommend dietary supplements: report of a survey Nutr J 2009 8 29 10.1186/1475-2891-8-29 19570197 PMC2714854

[B22] Life...supplemented consumer wellness program http://www.lifesupplemented.org/

[B23] Foote JA Murphy SP Wilkens LR Hankin JH Henderson BE Kolonel LN Factors associated with dietary supplement use among healthy adults of five ethnicities: the Multiethnic Cohort Study Am J Epidemiol 2003 157 10 888 897 10.1093/aje/kwg072 12746241 PMC2768595

[B24] CRN Consumer Survey on Dietary Supplements http://www.crnusa.org/prpdfs/CRNPR2009CRNConsumerSurvey_UsageConfidence.pdf

[B25] Adams KM Lindell KC Kohlmeier M Zeisel SH Status of nutrition education in medical schools Am J Clin Nutr 2006 83 4 941S 944S 16600952 10.1093/ajcn/83.4.941S PMC2430660

